# Urinary volatile organic compounds as potential non-invasive markers for childhood obesity

**DOI:** 10.1007/s11306-026-02494-6

**Published:** 2026-07-01

**Authors:** Adebowale Samuel Oyerinde, Jeremy Carson, Mallory Gibson, JackQuoia Baulding, Melissa Boersma, Robert A. Oster, Jeganathan Ramesh Babu, Thangiah Geetha

**Affiliations:** 1 Department of Nutritional Sciences, Auburn University, Auburn, AL USA; 2 Department of Biology, Auburn University, Auburn, AL USA; 3 Department of Chemistry, Auburn University, Auburn, AL USA; 4 Division of General Internal Medicine and Population Science, School of Medicine, University of Alabama at Birmingham, Birmingham, AL USA; 5 Department of Food, Nutrition, and Packaging Sciences, Clemson University, Clemson, SC USA

**Keywords:** Childhood obesity, Volatile organic compounds, Urine, Noninvasive biomarkers

## Abstract

**Background:**

Childhood obesity is a complex disease and adversely affects multiple organ systems and increases the risk for several health problems, including diabetes, cancer, atherosclerosis, and premature cardiovascular disease in adulthood. This study aimed to identify differences in volatile organic compounds (VOCs) between normal weight (NW) and overweight/obese (OW/OB) children, as well as between European American and African American children.

**Methods:**

A total of 159 children participated in this cross-sectional study. Urine samples were analyzed using solid-phase microextraction gas chromatography-mass spectrometry (SPME–GC–MS) following glucuronidase and sulfatase treatment to capture a broad range of urinary volatiles with diverse physicochemical properties. Multiple linear regression was used to examine associations between urinary VOCs and BMI z-scores, and to identify VOCs that differentiated NW from OW/OB children.

**Results:**

Sixty-five VOCs were identified as differing between NW and OW/OB children, with ten remaining significant predictors of obesity after adjusting for race and socioeconomic status. These compounds exhibited area under the curve values ranging from 0.503 and 0.737, with sensitivity and specificity above 0.60.

**Conclusions:**

Key VOCs, including benzaldehyde, furan, hexanal, 4-heptanone and 2-pentylfuran, were positively associated with BMI z-score and may contribute to the low-grade chronic inflammation characteristic of obesity. These findings highlight the potential of urinary VOCs as non-invasive biomarkers for childhood obesity.

**Supplementary Information:**

The online version contains supplementary material available at https://doi.org/10.1007/s11306-026-02494-6.

## Background

Obesity has emerged as one of the most significant global health challenges. In the United States, childhood obesity rates have risen steadily across all age groups since the late 1980s (Skinner et al., 2018). Children with obesity are at increased risk for metabolic disorders, including insulin resistance, type 2 diabetes, dyslipidemia, and nonalcoholic fatty liver disease (Alkhouri et al., 2015; Mijailovic et al., 2001). Although childhood obesity is widespread, it disproportionately affects children from lower socioeconomic status (SES) backgrounds (Lieb et al., 2009). Robert et al. reported that SES is a stronger predictor of obesity risk in children than race or ethnicity, underscoring the central role of economic disparities (Rogers et al., 2015). Similarly, Kim et al. found that Black and Hispanic children, as well as those from economically disadvantaged families, consistently had higher BMI values (Kim et al., 2024). Understanding how race, ethnicity, and SES interact to influence obesity risk is essential for designing targeted interventions that advance health equity (Mahmood et al., 2022).

Volatile organic compounds (VOCs), including alcohol, short-chain fatty acids, and saturated and unsaturated hydrocarbons, are generated endogenously through processes such as lipid peroxidation (LPO), protein oxidation, and gut microbial metabolism, and can also originate from exogenous environmental exposures (Cozzolino et al., 2017; Oyerinde et al., 2023). In obesity, increased production of reactive oxygen species (ROS) and activation of cytochrome P450 (CYP450) enzymes promote oxidative stress, contributing to disease-associated VOC signatures. These VOC patterns are relevant given the chronic low-grade inflammation characteristic of obesity and related metabolic disorders (Masenga et al., 2023; Oyerinde et al., 2023; Zhou et al., 2021).

While VOC analysis in exhaled breath has been studied more extensively in pediatric obesity (Alkhouri et al., 2015; Xu et al., 2023), research on urinary VOCs remains limited. Advanced analytical approaches, such as solid-phase microextraction (SPME), a solvent-free pre-concentration method that combines sampling, extraction, concentration, and sample introduction combined with capillary gas chromatography–mass spectrometry (GC–MS), have improved sensitivity and specificity for urinary VOC detection. This technology enables the identification of disease-related metabolic patterns.

The present study aimed to: (1) compare urinary VOC profiles between normal weight (NW) and overweight/obese (OW/OB) children, (2) identify VOCs associated with BMI z-scores while adjusting for race and SES, and (3) determine whether VOC profiles differ between African American (AA) and European American (EA) children. By characterizing multiple classes of urinary VOCs, this research seeks to provide new insights into the metabolic pathways and pathophysiological mechanisms underlying childhood obesity.

## Methods

### Study participants

This study recruited children aged 6–10 years from AA and EA ethnic groups. Written informed consent was obtained from parents or legal guardians, and assent was obtained from the children prior to enrollment. Demographic information, including date of birth, sex, race/ethnicity, maternal education, and family income, was recorded. Children were excluded if they had diabetes, cardiovascular disease, or sleep apnea, or if they were taking medications or antibiotics during the study period. The study protocol was approved by the Institutional Review Board of Auburn University (17-364 MR 1709) and Biological Use Authorization (1062).

### Anthropometric measurements and sample collection

Height and weight were measured following the World Health Organization (WHO) guidelines using a Tanita digital scale (WB-800H plus). Body Mass Index (BMI) was calculated according to the Centers for Disease Control and Prevention (CDC) growth charts, and participants were classified as normal weight (≥ 5th to ≤ 85th percentile), overweight (> 85th to ≤ 95th percentile), or obese (> 95th percentile) (Kuczmarski et al., 2002). To account for ongoing growth up to age 20, BMI z-scores were calculated using the WHO 2007 growth reference macro for SPSS. These smoothed percentile curves control for the confounding effects of age and sex during participant classification (Onis et al., 2007). Urine samples were collected during screening in sterile urine collection containers, aliquoted, and centrifuged at 2000 rpm for 10 min at 4 °C to remove particulates. The supernatant was stored at − 80 °C until analysis.

### Sample preparation and SPME procedure

VOC profiling was performed using the headspace SPME-GC-MS, with a divinylbenzene/carboxen/polydimethylsiloxane (DVB/CAR/PDMS) fiber (50/30 μm). Previous research study by Cozzolino et al. (2017) employed strong acid or base treatment to enhance volatile compounds in urine samples. In contrast, we adopted a mild enzymatic approach to enhance volatile compounds while improving reproducibility and preserving the native composition of urinary metabolites. The enzymatic solution was prepared by dissolving 100 µL each of β-glucuronidase and sulfatase enzyme in 10 mL of 0.15 M sodium citrate buffer (pH 4.6) containing L-ascorbic acid. For each 4 mL of urine sample, 500 µL of enzymatic solution and 5 µL of a stock solution containing three internal standards [2-β-pinene (BP), ethanol (EtOH) and 4-(Trifluoromethyl) benzenamine (TB)] were added to a 20 mL headspace vial. Vials were sealed with PTFE lined aluminum cap and incubated overnight at 37 °C, to allow VOCs to equilibrate into the headspace. The samples were then placed on a PAL system SPME-enabled autosampler, preincubated for 10 s at 60 °C, and agitated for 5 s. The fiber extracted VOCs for 60 min before being inserted into the GC inlet at 250 °C for 30 min. Fibers were conditioned prior to being used according to the manufacturer’s recommendations.

### GC–MS analysis

An Agilent 6890 N gas chromatograph coupled with 5975 mass selective detector was used for compound separation. VOCs were separated on a Restek Stabilwax-DA 30 m column (0.25 mm ID, 0.25 µm film thickness). The oven temperature was initially set at 35 °C and held for 5 min, followed by a ramp of 5 °C/min to 250 °C, with a final hold of 10 min, for a total run time of 58 min. A spitless injection mode was used with helium as the carrier gas at a flow rate of 1.6 mL/min. The MS transfer line was maintained at 260 °C, the source at 230 °C, and the quadrupole at 150 °C. Mass spectra were collected in fast-scan mode over the range 35–350 m/z. Chemstation software was used for instrument control, and data were exported as netCDF files for further processing.

GC–MS data were processed using MS-DIAL (versions 4.8 and 4.9.221218), a workflow previously validated by our laboratory (Oyerinde et al., 2025*).* Raw data in netCDF format were converted to ABF format using the Reifycs ABF converter integrated within MS-DIAL. The converted files were subsequently used to generate a project configured for hard ionization, centroid data acquisition, and positive ion mode analysis. Retention indices were calculated using an alkane standard mix analyzed on the same column. Data processing parameters were defined as follows: a minimum peak height threshold of 1000 amplitude, a deconvolution sigma window value of 0.5, and an electron ionization (EI) spectral cut-off of 10 amplitude. The retention index tolerance was set to 40, retention time tolerance to 0.5 min, m/z tolerance to 0.5, and the EI similarity threshold to 70%.

Peak alignment was conducted using a treated sample reference, with a retention time tolerance of 0.2 min, and gap filling was enforced. A peak count filter of 10% was applied, requiring features to be present in at least 10% of samples within a group. Features were excluded when their signal intensity was less than a fivefold difference relative to blank samples. Aligned datasets were exported as peak area matrices and further filtered based on ion abundances detected in blank samples before being saved as MSP files. These files were converted to CSV format using Microsoft Excel, where alignment ID, retention time, and metabolite names were concatenated into rows, and sample file names were assigned as column headers for downstream analysis using MetaboAnalyst.

Compound identification was performed using the automated mass spectral deconvolution and identification system (AMDIS) in combination with the National Institute of Standards and Technology (NIST) 2020 library. Most significant VOCs were further validated through comparison with authentic reference standards and confirmed through retention time and spectral matching to fully identify the compounds (Sumner et al., 2007). Reference standards, including furan, 2-pentanone, hexanal, 4-heptanone, 2-heptanone, 2-nonanone, 1-octen-3-ol, L-menthone, 2-ethyl-hexanol, 1,3-di-tert-butylbenzene, benzaldehyde, endo-borneol, dl-menthol, 1-nonanol, 2-decenal, terpenes, salicylaldoxime, geraniol, creosol, 3-methylphenol, 2-methylphenol, 2-methoxy-4-propylphenol, 2,4-di-tert-butylphenol, 4-vinylphenol, γ-dodecalactone, indole, homosalate, ethyl ester-9-octadecenoic acid, and *t*-butylhydroquinone, were obtained from VWR Chemicals. These standards were analyzed and compared with the corresponding urinary VOCs based on retention time and mass-to-charge ratios (m/z). Urinary VOC abundances were normalized to the total ion signal of each sample to generate relative peak percentages.

### Quality control of SPME–GC–MS analysis

Urine samples were analyzed over five consecutive days, with each run including internal standards (BP, EtOH, and TB) chosen for their absence in endogenous urine profiles. In this study, internal standards were included during data acquisition primarily for quality control purposes, including monitoring instrument stability, retention time reproducibility, signal drift, and optimization of MS-DIAL processing parameters. However, they were not used for signal normalization. Instead, VOC features were normalized to the total ion signal to account for global variation across samples. The internal standard peaks were excluded prior to normalization to avoid disproportionate influence on the total signal, given their relatively high and stable abundance compared to endogenous VOCs. This approach was selected to provide a straightforward global scaling across samples. A representative urine sample was analyzed repeatedly to assess system stability; corresponding chromatograms are shown in Fig. S1. Blank samples containing enzymatic digestion solutions, with or without internal standards, were also analyzed to monitor background signals and carryover. Retention time coefficients of variation (%CV) for internal standards in spiked water ranged from 0.3% to 2.3%, and peak area variability ranged from 13 to 38%, indicating acceptable reproducibility for biological VOC analysis. Principal component analysis (PCA) was used to visualize the distribution of VOCs among quality control and study samples (Fig. S2).

### Statistical analysis

In this cross-sectional study, associations between urinary VOCs and obesity were assessed using an integrated statistical framework. Descriptive statistics were used to compare baseline characteristics between NW and OW/OB children. Categorical variables were analyzed using the Chi-square test, whereas continuous variables were assessed using student’s t-test. Differences in VOC features between groups were assessed using the non-parametric Wilcoxon rank-sum test. Associations between VOCs and obesity outcomes were examined using multiple linear regression and binary logistic regression models. An unadjusted model was first fitted, followed by two adjusted models: Model 1 adjusted for race, and Model 2 adjusted for race, maternal education and family income. For VOCs exhibiting significant group differences, diagnostic performance was assessed by receiver operating characteristic (ROC) curve analysis, including calculation of the area under the curve (AUC), sensitivity and specificity. Partial least squares discriminant analysis (PLS–DA) and Random Forest model were applied to discriminate between classes and identify VOC signatures associated with sample groups. PLS-DA was used for both visualization and predictive modelling. Urinary data were auto scaled prior to analysis. The relationship between the input variables (X; urinary VOCs obtained by SPME–GC–MS) and the response variable (Y; class labels corresponding to NW and OW/OB children) was modelled to identify discriminative VOCs. Model validity was assessed by permutation testing (n = 100), in which the goodness-of-fit (R^2^ and Q^2^) of the original model was compared with that of models generated after random permutation of Y-class labels. Variables contributing to group discrimination were identified using VIP scores. In addition, random forest analysis was performed to identify urinary VOCs associated with obesity and to validate features identified by multivariate linear regression analysis. Statistical significance was defined as *P* < 0.05, and an AUC value greater than 0.50 (α = 0.05) was considered indicative of discriminatory capability. All statistical analyses were conducted using MetaboAnalyst (version 6.0) (Chong et al., 2018), SPSS (version 29.0.0.0), and R software.

## Results

The characteristics of the study participants are summarized in Table 1. A total of 159 children were included, comprising 98 NW and 61 OW/OB participants. Obesity-related measures, including body weight, BMI, and BMI z-score, were significantly higher in OW/OB compared to NW children.

**Table 1:** General characteristics of the study population

Baseline data	NW (n = 98)	OW/OB (n = 61)	*P*-value
Age (years), mean ± SD	8.27 ± 1.45	8.66 ± 1.38	0.085
**Sex (%)**			0.792
Male	51.02	47.54	
Female	48.98	52.46	
**Race (%)**			0.968
European American	54.08	55.74	
African American	45.92	44.26	
Obesity measures (mean ± SD)			
Weight (kg)	27.51 ± 6.48	39.84 ± 12.23	< 0.001
Height (cm)	130.41 ± 11.47	135.66 ± 11.42	0.006
BMI (kg/m^2^)	15.94 ± 1.45	21.20 ± 2.98	< 0.001
BMI *z*-score	− 0.062 ± 0.79	1.98 ± 0.58	< 0.001
**Maternal education (%)**			0.934
High school or less	24.49	22.95	
Associate degree	22.45	26.23	
Bachelor’s degree	23.47	24.59	
Graduate degree	29.59	26.23	
**Family income (%)**			0.537
< $25,000	33.67	22.95	
$25,001–$50,000	15.31	18.03	
$50,001–$75,000	14.29	14.75	
> $75,001	36.73	44.26	

*NW* normal weight, *OW/OB* overweight/obese, *BMI* body mass index, *WC* waist circumference, *WHtR* waist height ratio

The identified metabolites represented a wide range of chemical classes, including ketones, hydrocarbons, aldehydes, alcohols, acids, terpenes, furans, alkanals, nitrogen- and sulfur-containing compounds, esters, and ethers. A total of 721 metabolite features, each defined as a distinct signal characterized by a specific mass-to-charge ratio and retention time representing a putative metabolite in the dataset, were detected in samples from both NW and OW/OB children.

**Fig. 1:**
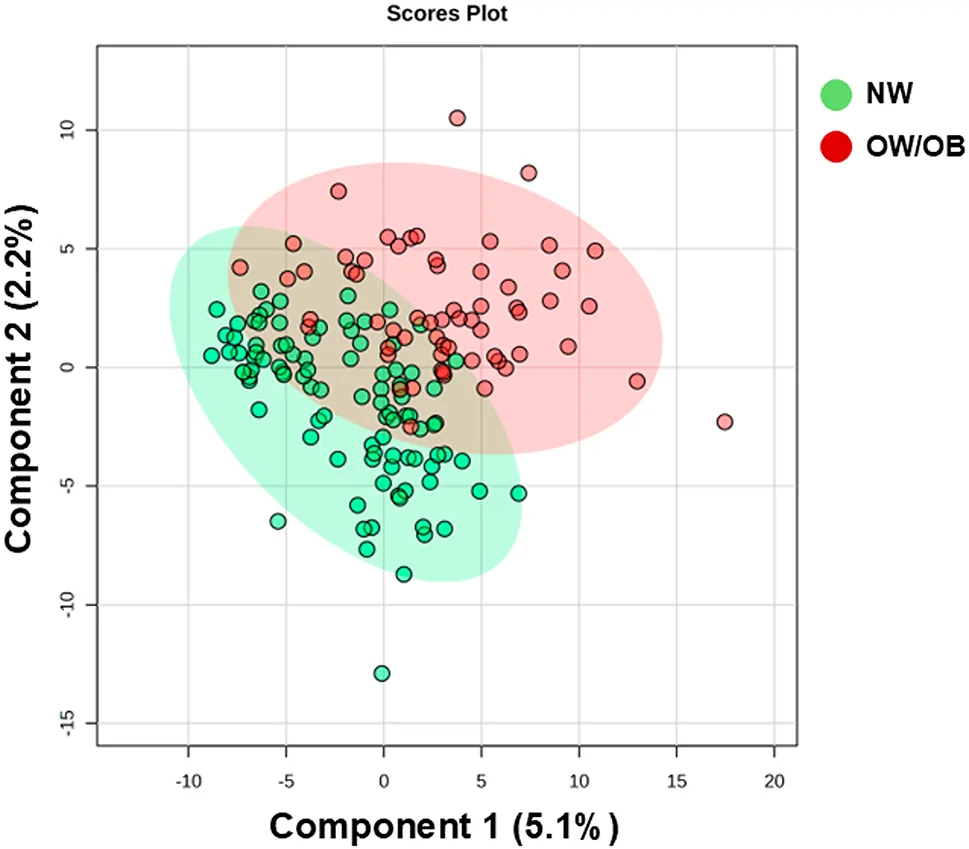
Partial least squares-discriminant analysis (PLS-DA) score scatter plot based on the data set of the overall participants’ urine obtained under glucuronidase and sulfatase conditions. NW children are indicated with green circles, whereas OW/OB subjects are represented by red circles. Components 1 and 2 are the first two latent variables that explain the highest variance while maximizing class separation

**Fig. 2:**
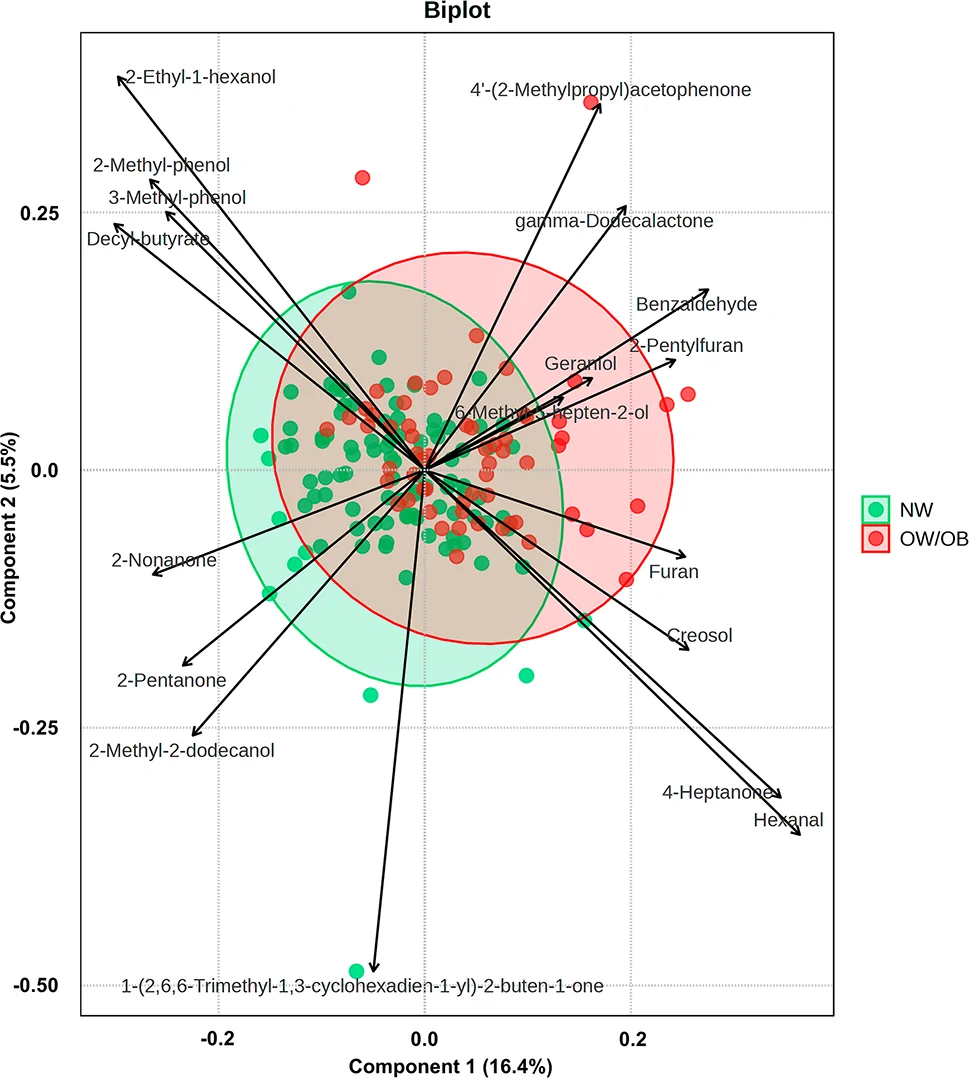
Partial least square-discriminant analysis (PLS-DA) biplot based on the significant urinary VOCs associated with obesity. NW children are indicated with green circles whereas OW/Ob subjects with red circles. The association was observed using multiple linear regression. Components 1 and 2 are the first two latent variables that explain the highest variance while maximizing class separation

PLS-DA was performed to identify urinary VOCs responsible for discriminating NW from OW/OB individuals. The representative score plot (Fig. 1) demonstrated a visualized separation between NW and OW/OB groups, indicating distinct urinary VOC profiles. Model construction was based on VOCs that were statistically significant between groups following non-parametric Wilcoxon testing. The corresponding biplot (Fig. 2) illustrated the contribution of individual variables to class separation. Model validation by permutation testing (Fig. S4) provide strong evidence that model performance is not due to random structure in the data and is therefore considered robust in PLS-DA reporting and confirmed that the observed discrimination was statistically significant (*P* < 0.02, 2/100). The PLS-DA model exhibited moderate discriminatory performance, yielding a highest classification accuracy of 0.692, indicative of modest separation between groups. Variables contributing to group discrimination were identified using VIP scores, with several VOCs exceeding the threshold of 1. These included benzaldehyde, hexanal, 2-nonanone, 4-heptanone, 2-pentanone, furan, 2-pentylfuran, decyl-butyrate, gamma-dodecalactone, 2-methyl-2-dodecanol, all of which showed consistent importance across components (Fig. S5). The Random Forest model identifies the most important VOCs contributing to classification between the study groups based on mean decrease in accuracy (Fig. S3).

The Wilcoxon rank-sum test identified 39 VOCs that were significantly higher in OW/OB compared to NW children (Table 2) and 26 VOCs that were significantly higher in NW compared to OW/OB children (Table 3). Detailed information for all identified urinary VOCs, including fragment ion m/z values, highest-abundance peaks, NIST spectral match, the experimentally computed retention index (Kovats index) using an alkane standard mix analyzed on the same column, and frequency of occurrence, is provided in Supplementary Table 1.

**Table 2:** Urinary VOCs that are significantly higher in overweight/obese compared to normal weight children

VOCs	AUC	AUC ROC (95% CI)	*P*-value	Sensitivity	Specificity
Furan	0.602	0.507–0.698	0.030	0.44	0.79
2-Methyl propanal	0.611	0.522–0.700	0.019	0.70	0.5
2-Ethyl-5-methyl-furan	0.625	0.534–0.716	0.008	0.72	0.55
Hexanal	0.650	0.562–0.737	0.002	0.64	0.66
4-Heptanone	0.597	0.504–0.689	0.041	0.69	0.57
2-Heptanone	0.595	0.507–0.683	0.045	0.79	0.44
2-Pentyl-furan	0.635	0.547–0.724	0.004	0.62	0.61
Allyl-isothiocyanate	0.614	0.525–0.703	0.016	0.69	0.54
2-Octanone	0.594	0.507–0.682	0.046	0.95	0.27
2-Methyl-2-decanol	0.611	0.521–0.700	0.019	0.87	0.38
1,3,5-Undecatriene	0.586	0.493–0.680	0.068	0.28	0.90
6-Methyl-5-hepten-2-ol	0.600	0.510–0.691	0.034	0.56	0.66
5-Methyl-2-(1-methylethyl)- cis- cyclohexanone	0.605	0.515–0.694	0.027	0.74	0.46
L-Menthone	0.617	0.529–0.705	0.013	0.74	0.5
4-Pentyl-phenol	0.663	0.577–0.749	0.001	0.69	0.59
3-Hydroxy-4-methoxybenzaldehyde	0.596	0.503–0.688	0.043	0.41	0.80
3-Amino-5-t-butylisoxazole	0.622	0.533–0.710	0.010	0.52	0.72
Benzaldehyde	0.653	0.566–0.740	0.001	0.72	0.61
Salicylaldoxime	0.602	0.511–0.693	0.031	0.74	0.48
Geraniol	0.626	0.538–0.715	0.007	0.62	0.63
2-Methyl-, 3-hydroxy-2,2,4-trimethylpentyl propanoic ester	0.614	0.524–0.705	0.015	0.33	0.88
trans-Farnesol	0.611	0.521–0.701	0.019	0.77	0.46
alpha-Calacorene	0.635	0.0547–0.723	0.004	0.74	0.51
4ʹ-(2-Methylpropyl) acetophenone	0.620	0.530–0.709	0.011	0.64	0.56
Creosol	0.594	0.502–0.687	0.046	0.34	0.84
p-Mentha-1,8-dien-7-ol	0.608	0.517–0.699	0.022	0.74	0.48
p-Cresol	0.616	0.528–0.704	0.014	0.85	0.40
4,4-Dimethyl-6-hydroxy-3,4-dihydrocoumarin	0.593	0.504–0.682	0.049	0.92	0.28
2,4-Di-tert-butylphenol	0.606	0.516–0.695	0.025	0.52	0.68
3,7,11-Trimethyl-2,6,10-dodecatrienal	0.599	0.508–0.691	0.035	0.46	0.76
2-Methoxy-4-(1-propenyl)-phenol	0.594	0.504–0.684	0.046	0.44	0.76
gamma-Dodecalactone	0.614	0.520–0.708	0.016	0.69	0.60
Indole	0.618	0.527–0.709	0.013	0.64	0.59
Homosalate	0.597	0.504–0.690	0.040	0.57	0.62
9-Octadecenoic acid, ethyl ester	0.600	0.511–0.689	0.035	0.80	0.42
2-(4-(But-2-yl) phenyl) propanoic acid	0.612	0.522–0.701	0.018	0.56	0.66
Benzoic acid, 2-hydroxy-, phenylmethyl ester	0.607	0.518–0.695	0.024	0.62	0.63
Octadecamethyl-cyclononasiloxane	0.613	0.524–0.702	0.017	0.48	0.74
t-Butylhydroquinone	0.612	0.525–0.700	0.017	0.74	0.47

*VOCs* volatile organic compounds, *AUC* area under the curve, *ROC* receiver operating characteristic, *CI* confidence interval

**Table 3:** Urinary VOCs that are significantly higher in normal weight compared to the overweight/obese children

VOCs	AUC	AUC ROC (95% CI)	*P*-value	Sensitivity	Specificity
2-Pentanone	0.620	0.531–0.710	0.011	0.62	0.56
2-Ethyl-1-pyrroline	0.597	0.507–0.686	0.041	0.79	0.42
trans-1-Phenyl-1-pentene	0.618	0.530–0.707	0.012	0.89	0.34
7-Ethyl-1,3,5-cycloheptatriene	0.596	0.508–0.684	0.042	0.74	0.47
2-Nonanone	0.593	0.505–0.681	0.049	0.97	0.24
1,3-Bis(1,1-dimethylethyl)-benzene	0.600	0.508–0.692	0.034	0.74	0.5
1-Octen-3-ol	0.596	0.505–0.686	0.043	0.62	0.61
5-Ethyl-6-methyl-3E-hepten-2-one	0.630	0.542–0.717	0.006	0.82	0.44
2-Ethyl-1-hexanol	0.604	0.515–0.692	0.028	0.80	0.43
2-Methyl-2-dodecanol	0.606	0.518–0.695	0.024	0.87	0.33
dl-Menthol	0.598	0.503–0.693	0.037	0.62	0.65
endo-Borneol	0.614	0.525–0.704	0.016	0.61	0.69
1-Nonanol	0.606	0.516–0.696	0.025	0.82	0.38
2-Decenal	0.619	0.528–0.710	0.012	0.62	0.61
alpha-Terpineol	0.598	0.508–0.687	0.039	0.79	0.45
p-Mentha-1,5-dien-8-ol	0.601	0.512–0.690	0.032	0.72	0.5
1-(2,6,6-Trimethyl-1,3-cyclohexadien-1-yl)- 2-buten-1-one	0.598	0.509–0.687	0.037	0.82	0.42
Decyl-butyrate	0.609	0.520–0.697	0.022	0.89	0.37
3-Methyl-phenol	0.612	0.523–0.702	0.018	0.66	0.60
2-Methyl-phenol	0.593	0.504–0.682	0.050	0.80	0.46
2-Methoxy-4-propyl-phenol	0.606	0.517–0.695	0.025	0.77	0.43
1,6-Dimethyl-4-(1-methylethyl)- naphthalene	0.599	0.508–0.689	0.037	0.75	0.47
4-Vinylphenol	0.601	0.508–0.694	0.033	0.49	0.76
Chloroxylenol	0.624	0.535–0.712	0.009	0.69	0.55
2,7-Dimethyl-2,6-octadien-4-ol	0.621	0.531–0.711	0.010	0.59	0.70
1-Dimethylphenylsilyloxy-4-methoxybenzene	0.601	0.509–0.694	0.032	0.67	0.59

*VOCs* volatile organic compounds, *AUC* area under the curve, *ROC* receiver operating characteristic, *CI* confidence interval

To assess the association between VOCs and BMI z-score, we conducted multiple linear regression and binary logistic regression analyses using VOCs that significantly differed between NW and OW/OB groups (Tables 2 and 3). In the unadjusted linear regression model, nine VOCs were significantly and positively associated with BMI z-score. Benzaldehyde showed the strongest positive association, followed by 4′-(2-methylpropyl) acetophenone, furan, 6-methyl-5-hepten-2-ol, hexanal, 4-heptanone, 2-pentylfuran, geraniol, and creosol. Four VOCs were significantly and inversely associated with BMI z-score: 2-methyl-2-dodecanol, 1-(2,6,6-trimethyl-1,3-cyclohexadien-1-yl)-2-buten-1-one, decyl-butyrate, and 2-methylphenol. In Model 1 (adjusted for race), benzaldehyde remained the strongest positive predictor, followed by 4′-(2-methylpropyl) acetophenone, furan, and 4-heptanone. Additional positive associations were observed for 6-methyl-5-hepten-2-ol, hexanal, geraniol, and creosol. The strongest negative association was seen with 2-methylphenol, with similar negative trends for 1-(2,6,6-trimethyl-1,3-cyclohexadien-1-yl)-2-buten-1-one, decyl-butyrate, and 2-methyl-2-dodecanol. In Model 2 (adjusted for race, family income, and maternal education), ten VOCs were significantly and positively associated with obesity, including γ-dodecalactone, which had not been significant in previous models, suggesting that socioeconomic factors may influence obesity-related metabolic patterns. Three VOCs, 1-(2,6,6-trimethyl-1,3-cyclohexadien-1-yl)-2-buten-1-one, 2-methyl-2-dodecanol, and 3-methylphenol were significantly and inversely associated with BMI z-score in the fully adjusted model (Table 4).

**Table 4:** The association between urinary VOCs and BMI z-score using multiple linear regression

VOCs	Unadjusted		Model 1		Model 2	
	β (LB-UB)	*P* value	β (LB-UB)	*P* value	β (LB-UB)	*P* value
Furan	0.224 (5.190–27.919)	0.005	0.223 (5.113–27.908)	0.005	0.207 (3.991–26.558)	0.008
6-Methyl-5-hepten-2-ol	0.172 (0.972–19.569)	0.031	0.173 (1.028–19.682)	0.030	0.171 (1.071–19.378)	0.029
Hexanal	0.166 (2.007–62.204)	0.037	0.168 (2.278–62.675)	0.035	0.160 (1.099–60.714)	0.042
4-Heptanone	0.163 (0.008–0.358)	0.041	0.176 (0.019–0.376)	0.030	0.126 (− 0.041–0.324)	0.127
2-Pentylfuran	0.160 (0.423–29.349)	0.044	0.161 (0.422–29.424)	0.044	0.149 (− 0.451–28.087)	0.058
Benzaldehyde	0.249 (3.643–15.179)	0.002	0.249 (3.627–15.193)	0.002	0.231 (2.901–14.591)	0.004
Geraniol	0.164 (0.636–22.908)	0.038	0.163 (0.387–22.890)	0.043	0.163 (0.657–22.734)	0.038
4ʹ-(2-Methylpropyl) acetophenone	0.225 (14.828–78.845)	0.004	0.232 (16.013–80.522)	0.004	0.238 (18.088–81.257)	0.002
Creosol	0.163 (0.415–17.529)	0.040	0.161 (0.226–17.509)	0.044	0.173 (1.054–18.006)	0.028
gamma-Dodecalactone	0.134 (− 3.606–46.763)	0.093	0.137 (− 3.209–47.402)	0.087	0.170 (2.540–52.372)	0.031
1-(2,6,6-Trimethyl-1,3-cyclohexadien-1-yl)2-buten-1-one	− 0.157 (− 4.618 to − 0.019)	0.048	− 0.164 (− 4.750 to − 0.104)	0.041	− 0.163 (− 4.695 to − 0.134)	0.038
2-Methyl-2-dodecanol	− 0.161 (− 5.857 to − 0.109)	0.042	− 0.162 (− 5.883 to − 0.119)	0.041	− 0.155 (− 5.687 to − 0.023)	0.048
Decyl-butyrate	− 0.164 (− 0.625 to − 0.017)	0.039	− 0.163 (− 0.624 to − 0.014)	0.040	− 0.146 (− 0.586 to 0.016)	0.063
3-Methyl-phenol	− 0.155 (− 0.042 to 0.000)	0.051	− 0.155 (− 0.042–0.000)	0.052	− 0.156 (− 0.042 to 0.000)	0.046
2-Methyl-phenol	− 0.169 (− 3.899 to − 0.169)	0.033	− 0.168 (3.890 to − 0.136)	0.036	− 0.149 (− 3.643 to 0.072)	0.059

Unadjusted: model without any covariates; model 1: adjusted by race; model 2: adjusted by race, family income and maternal education

*β* standardized coefficients, *LB* lower bound, *UB* upper bound

Binary logistic regression using obesity status (NW vs. OW/OB) as the outcome showed that, in the unadjusted model, eight VOCs were significantly and positively associated with BMI z-score. The strongest predictors were 2-pentylfuran, benzaldehyde, furan, and 4′-(2-methylpropyl) acetophenone, followed by hexanal, 4-heptanone, and γ-dodecalactone. Four VOCs were significantly and inversely associated with obesity, with decyl-butyrate showing the strongest negative association, followed by 2-methyl-2-dodecanol, 2-nonanone, and 3-methylphenol. In Model 1 (adjusted for race), significance levels were unchanged. In Model 2 (adjusted for race, family income, and maternal education), creosol emerged as an additional significant positive predictor (Supplementary Table 2). These associations were visualized using a PLS-DA biplot (Fig. 2) and validated with Random Forest analysis (Fig. S3).

ROC analysis was performed to evaluate the predictive potential of urinary VOCs for childhood obesity. AUC, *P*-values, sensitivity, and specificity were calculated for significant VOCs. Benzaldehyde demonstrated the highest predictive accuracy, followed by hexanal, 2-pentylfuran, geraniol, and γ-dodecalactone. Additional VOCs with predictive value included 4′-(2-methylpropyl) acetophenone and furan. Creosol exhibited high specificity (84%) despite a lower AUC (0.594). Several VOCs were inversely associated with obesity, including 2-ethyl-1-hexanol, decyl-butyrate, 3-methylphenol, 2-methyl-2-dodecanol, and 1-(2,6,6-trimethyl-1,3-cyclohexadien-1-yl)-2-buten-1-one (Table 5).

**Table 5:** Urinary VOCs significantly associated with BMI z-score after adjusted for race and SES

VOCs	Direction	AUC	AUC ROC (95% CI)	*P* value	Sensitivity	Specificity
Furan	OW/OB > NW	0.602	0.507–0.698	0.030	0.44	0.79
6-Methyl-5-hepten-2-ol	OW/OB > NW	0.600	0.510–0.691	0.034	0.56	0.66
Hexanal	OW/OB > NW	0.650	0.562–0.737	0.002	0.64	0.66
4 Heptanone	OW/OB > NW	0.597	0.504–0.689	0.041	0.69	0.57
2-Pentylfuran	OW/OB > NW	0.635	0.546–0.724	0.004	0.62	0.61
Benzaldehyde	OW/OB > NW	0.653	0.566–0.740	0.001	0.72	0.61
Geraniol	OW/OB > NW	0.626	0.538–0.715	0.007	0.62	0.63
gamma-Dodecalactone	OW/OB > NW	0.614	0.520–0.708	0.016	0.69	0.60
4ʹ-(2-Methylpropyl) acetophenone	OW/OB > NW	0.620	0.530–0.709	0.011	0.64	0.56
Creosol	OW/OB > NW	0.594	0.502–0.687	0.046	0.34	0.84
2 Ethyl-1-hexanol	NW > OW/OB	0.630	0.542–0.717	0.006	0.82	0.44
2-Nonanone	NW > OW/OB	0.593	0.505–0.681	0.049	0.97	0.24
2-Methyl-2-dodecanol	NW > OW/OB	0.606	0.518–0.695	0.024	0.87	0.33
1-(2,6,6-Trimethyl-1,3-cyclohexadien-1-yl)-2-buten-1-one	NW > OW/OB	0.598	0.509–0.687	0.037	0.82	0.42
Decyl-butyrate	NW > OW/OB	0.609	0.520–0.697	0.022	0.89	0.37
3-Methyl-phenol	NW > OW/OB	0.612	0.523–0.702	0.018	0.66	0.60
2-Methyl-phenol	NW > OW/OB	0.593	0.504–0.682	0.050	0.80	0.46

*NW* normal weight, *OW/OB* overweight/obese, *AUC* area under the curve, *ROC* receiver operating characteristic curve, *CI* confidence interval

The Wilcoxon rank-sum test was used to assess association of VOC within EA and AA groups. Overall, VOCs such as furan, hexanal, 4-heptanone, 2-pentylfuran, benzaldehyde, and γ-dodecalactone were significantly higher in OW/OB children, whereas 2-ethyl-1-hexanol, 2-nonanone, 2-methyl-2-dodecanol, decyl-butyrate, and 3-methylphenol were significantly lower.

Among EA participants, hexanal, 4-heptanone, creosol, 4′-(2-methylpropyl) acetophenone, and benzaldehyde were significantly higher in OW/OB, while 2-ethyl-1-hexanol was more prevalent in NW children. In AA participants, 6-methyl-5-hepten-2-ol, 2-pentylfuran, and geraniol were significantly higher in OW/OB, whereas 3-methylphenol and 2-methyl-2-dodecanol were significantly lower.

When comparing racial patterns, among OW/OB children, hexanal, 4-heptanone, creosol, 4′-(2-methylpropyl) acetophenone, and benzaldehyde were significantly higher in EA, while 6-methyl-5-hepten-2-ol, 2-pentylfuran, and geraniol was significantly higher in AA participants. Among NW children, 2-ethyl-1-hexanol was significantly higher in EA, while 3-methylphenol and 2-methyl-2-dodecanol were significantly higher in AA participants (Table 6).

**Table 6:** Urinary VOCs significantly different between normal-weight and overweight/obese children from European American and African American ethnicity

VOCs	Total				EA				AA			
	NW mean (C1)	OW/OB mean (C1)	*P* value	Direction	NW mean (C1)	OW/OB mean (C1)	*P* value	Direction	NW mean (C1)	OW/OB mean (C1)	*P* value	Direction
Furan	0.023 (0.020–0.026)	0.030 (0.025–0.035)	0.03	OW/OB > NW	0.023 (0.019–0.027)	0.029 (0.023–0.036)	0.099	OW/OB > NW	0.023 (0.019–0.028)	0.031 (0.023–0.039)	0.171	OW/OB > NW
6-Methyl-5-hepten-2-ol	0.005 (0.001–0.008)	0.008 (0.0019–0.0150)	0.034	OW/OB > NW	0.007 (0.0008–0.013)	0.006 (− 0.003–0.016)	0.629	NW > OW/OB	0.002 (0.001–0.003)	0.011 (0.002–0.020)	0.004	OW/OB > NW
Hexanal	0.004 (0.003–0.005)	0.007 (0.0049–0.0089)	0.002	OW/OB > NW	0.004 (0.003–0.006)	0.007 (0.005–0.01)	0.004	OW/OB > NW	0.004 (0.003–0.007)	0.006 (0.003–0.009)	0.114	OW/OB > NW
4-Heptanone	0.931 (0.760–1.101)	1.359 (1.011–1.706)	0.041	OW/OB > NW	0.965 (0.718–1.213)	1.777 (1.211–2.343)	0.006	OW/OB > NW	0.891 (0.649–1.132)	0.832 (0.584–1.081)	0.963	NW > OW/OB
2-Pentylfuran	0.007 (0.005–0.009)	0.013 (0.009–0.017)	0.004	OW/OB > NW	0.008 (0.005–0.011)	0.012 (0.007–0.018)	0.188	OW/OB > NW	0.006 (0.003–0.009)	0.014 (0.008–0.020)	0.007	OW/OB > NW
Benzaldehyde	0.007 (0.005–0.010)	0.024 (0.012–0.036)	0.043	OW/OB > NW	0.005 (0.003–0.007)	0.027 (0.009–0.045)	0.040	OW/OB > NW	0.010 (0.004–0.016)	0.020 (0.003–0.037)	0.548	OW/OB > NW
Geraniol	0.004 (0.003–0.005)	0.009 (0.002–0.015)	0.007	OW/OB > NW	0.004 (0.002–0.005)	0.004 (0.003–0.006)	0.196	OW/OB > NW	0.004 (0.002–0.007)	0.014 (− 0.001–0.030)	0.007	OW/OB > NW
gamma-Dodecalactone	0.005 (0.004–0.006)	0.008 (0.005–0.010)	0.016	OW/OB > NW	0.005 (0.004–0.007)	0.008 (0.005–0.012)	0.152	OW/OB > NW	0.004 (0.003–0.006)	0.007 (0.004–0.010)	0.039	OW/OB > NW
4ʹ-(2-Methylpropyl) acetophenone	0.0009 (0.0007–0.001)	0.003 (0.0008–0.006)	0.011	OW/OB > NW	0.0009 (0.0007–0.0013)	0.005 (0.0003–0.009)	0.022	OW/OB > NW	0.0008 (0.0005–0.001)	0.001 (0.0004–0.003)	0.215	OW/OB > NW
Creosol	0.005 (0.001–0.008)	0.012 (0.005–0.0197)	0.046	OW/OB > NW	0.002 (0.0008–0.0036)	0.01 (0.0005–0.020)	0.017	OW/OB > NW	0.008 (0.0006–0.015)	0.015 (0.004–0.026)	0.764	OW/OB > NW
2 Ethyl-1-hexanol	0.092 (0.068–0.117)	0.052 (0.019–0.086)	0.006	NW > OW/OB	0.111 (0.073–0.149)	0.062 (0.004–0.121)	0.015	NW > OW/OB	0.071 (0.042–0.099)	0.040 (0.019–0.062)	0.224	NW > OW/OB
2-Nonanone	0.055 (0.030–0.081)	0.005 (0.0003–0.010)	0.049	NW > OW/OB	0.076 (0.035–0.117)	0.003 (0.002–0.004)	0.087	NW > OW/OB	0.031 (0.006–0.057)	0.008 (− 0.003–0.019)	0.157	NW > OW/OB
2-Methyl-2-dodecanol	0.048 (0.033–0.063)	0.023 (0.011–0.035)	0.024	NW > OW/OB	0.045 (0.025–0.065)	0.024 (0.009–0.039)	0.302	NW > OW/OB	0.051 (0.028–0.075)	0.022 (0.001–0.042)	0.023	NW > OW/OB
1-(2,6,6-Trimethyl-1,3-cyclohexadien-1-yl)-2-buten-1-one	0.032 (0.012–0.052)	0.012 (0.003–0.022)	0.037	NW > OW/OB	0.018 (0.009–0.027)	0.009 (0.002–0.017)	0.260	NW > OW/OB	0.048 (0.005–0.092)	0.016 (− 0.004–0.036)	0.075	NW > OW/OB
Decyl-butyrate	0.463 (0.324–0.602)	0.227 (0.104–0.349)	0.022	NW > OW/OB	0.499 (0.287–0.710)	0.224 (0.062–0.387)	0.096	NW > OW/OB	0.421 (0.242–0.60)	0.230 (0.031–0.429)	0.125	NW > OW/OB
3-Methyl-phenol	12.397 (10.572–14.223)	9.321 (7.127–11.514)	0.018	NW > OW/OB	12.365 (9.666–15.063)	9.762 (6.450–3.073)	0.143	NW > OW/OB	12.436 (9.930–14.941)	8.765 (5.846–11.684)	0.045	NW > OW/OB
2-Methyl-phenol	0.141 (0.119–0.163)	0.109 (0.087–0.132)	0.050	NW > OW/OB	0.149 (0.119–0.180)	0.116 (0.083–0.150)	0.136	NW > OW/OB	0.131 (0.098–0.164)	0.101 (0.071–0.131)	0.202	NW > OW/OB

*NW* normal weight, *OW/OB* overweight/obese, *EA* European American, *AA* African American, *CI* confidence interval

## Discussion

The key findings of this study are OW/OB children exhibit a distinct urinary VOC profile compared to NW; SES is a significant covariate in obesity prevalence; and VOC profiles differ by race.

A total of 721 features were detected in urine from both NW and OW/OB children under β-glucuronidase and sulfatase treatment conditions. Changes in urinary VOC patterns are often linked to gut microbial dysbiosis, a condition associated with obesity and other gastrointestinal disorders (Cozzolino et al., 2017; Turnbaugh et al., 2009). Obesity is known to correlate with reduced microbial diversity and altered bacterial gene representation (Chierico et al., 2017; Cozzolino et al., 2017). Additionally, in obese individuals, VOCs alterations are related to oxidative stress, in which overproduction of reactive oxygen species in adipose tissue leads to LPO and cellular damage (Manna & Jain, 2015; Oyerinde et al., 2023, 2025; Swiatkiewicz et al., 2023). VOCs can also arise from exogenous exposures, such as diet and environmental factors (Cozzolino et al., 2017; McGraw et al., 2024; Mendy et al., 2022).

Several urinary VOCs identified in this study may serve as potential biomarkers for childhood obesity. Benzaldehyde emerged as one of the strongest predictors. Endogenously, benzaldehyde can be formed through multiple metabolic pathways, including LPO, carbohydrate metabolism, ascorbate autoxidation, amine oxidase activity, cytochrome P450 enzyme activity, and myeloperoxidase-catalyzed reactions (O’Brien et al., 2005). Mao et al. reported elevated serum benzaldehyde concentrations in the NHANES dataset, in which 68.89% of participants were classified as overweight or obese (Mao et al., 2023). Other studies suggest that benzaldehyde may be a biomarker for oxidative stress, cardiovascular disease, and cancer (Silva et al., 2011).

Our findings also showed elevated levels of furan and 2-pentylfuran in OW/OB children. Furan is generated through LPO of polyunsaturated fatty acids (PUFAs), which produces reactive aldehydes that cyclize into furan structures (Peterson, 2013). Microbial metabolism and dietary sources further contribute to furan formation (EPCF Chain, 2017; Gates et al., 2014; Kalisch et al., 2024). Hexanal, another VOC upregulated in OW/OB children and positively associated with BMI z-scores, is a secondary product of linoleic acid oxidation and an important mediator in oxidative stress regulation (Elisia & Kitts, 2011; Zajdel et al., 2007). Under oxidative stress, free radicals promote LPO and subsequent hexanal production (Elisia & Kitts, 2011). Hexanal detoxification is mediated by aldehyde dehydrogenase (ALDH) enzymes (Rodriguez-Zavala et al., 2019), and ALDH inhibition can lead to aldehyde accumulation, increasing the risk of mutagenesis and carcinogenesis (Cho et al., 2017). Additionally, hexanal has been implicated as a growth-regulating factor and signaling molecules in obesity-related metabolic dysregulation (Cho et al., 2017).

Ketones also appeared to play a biological role, with significant differences observed between NW and OW/OB children. We found elevated levels of 4-heptanone, γ-dodecalactone, and 4′-(2-methylpropyl) acetophenone, along with reduced levels of 2-nonanone and 1-(2,6,6-trimethyl-1,3-cyclohexadien-1-yl)-2-buten-1-one in OW/OB children. Ketones are a major class of urinary metabolites and have been linked to gut microbial dysbiosis in obesity (Chierico et al., 2017; Cozzolino et al., 2017). 4-Heptanone, a common urinary volatile, can result from in vivo decarboxylation of 3-oxo-2-ethylhexanoic acid, a compound derived from plasticizers (Riccio et al., 2022; Walker & Mills, 2001). Elevated 4-heptanone levels have been reported in diabetes (Liebich & Huesgen, 1976), kidney disease and various cancers (Riccio et al., 2022; Silva et al., 2019; Wang et al., 2016).

Regarding alcohol, we observed significantly higher levels of 6-methyl-5-hepten-2-ol, geraniol, and creosol in OW/OB compared to NW children. Geraniol, a natural compound found in fruits and herbs, is widely used as a flavoring agent, and higher urinary levels could reflect increased consumption of processed foods containing geraniol (Chand et al., 2023; Owsienko et al., 2024). 6-Methyl-5-hepten-2-ol is produced via LPO, driven by obesity-induced oxidative stress (Cozzolino et al., 2017). These results are consistent with previous studies indicating that gut microbial species, such as Bacteroides, can produce alcohols, potentially explaining the abundance of some alcoholic VOCs in OW/OB children (Zhu et al., 2013). Creosol, a methoxyphenol, is likely produced by gut microbes metabolizing lignin or polyphenols from plant-rich diets (Zhang & Dang, 2022). Conversely, 3-methyl-phenol and 2-methyl-phenol were more abundant in NW children, which may reflect higher protein diet, as microbial metabolism of tyrosine yields phenolic compounds (Poesen et al., 2015).

Overall, we identified benzaldehyde, furan, hexanal, 4-heptanone, and 2-pentylfuran as key VOCs positively associated with BMI z-score, supporting previous research that specific urinary volatile compounds contribute to the metabolic signature of adiposity (Alkhouri et al., 2015; Elliott et al., 2015). Furthermore, our data indicates that VOC profiles vary by race, suggesting that both metabolic and environmental factors may influence obesity risk differently in EA and AA children (Caprio et al., 2008; Walker et al., 2012).

Limitations include the absence of dietary and environmental exposure data and the cross-sectional study design, which precludes causal inference and limits the ability to determine whether several identified VOC biomarkers originated from dietary intake, environmental exposures, or other external sources. The urine samples analyzed in this study are inherently heterogeneous, as they were obtained from children. Accordingly, sensitivity and specificity estimates derived from single time-point urine samples are unlikely to represent optimal performance. Future studies will address these limitations through the inclusion of larger cohorts, implementation of controlled dietary conditions, and longitudinal sample collection. Nonetheless, a major strength is the use of non-invasive urine sampling, offering a simple and practical alternative to invasive diagnostic methods in pediatric populations.

This study identifies distinct urinary VOC profiles in OW/OB versus NW children, with compounds such as benzaldehyde, furan, hexanal, 4-heptanone, and 2-pentylfuran positively associated with BMI z-scores. Race and socioeconomic status (SES) influenced obesity risk, highlighting urinary VOCs as promising non-invasive biomarkers for early detection and understanding of childhood obesity.

## Supplementary Information

Below is the link to the electronic supplementary material.

Supplementary file1 (TIF 143 kb)

Supplementary file2 (TIF 200 kb)

Supplementary file3 (TIF 124 kb)

Supplementary file4 (TIF 111 kb)

Supplementary file5 (TIF 116 kb)

Supplementary file6 (DOCX 75 kb)

Supplementary file7 (DOCX 22 kb)

## Data Availability

All data generated during this study are included in this published article (and its Supplementary Information files).

## Abbreviations

VOC: Volatile organic compounds
BMI: Body mass index
SES: Socioeconomic status
NW: Normal weight
OW/OB: Overweight/obese
EA: European American
AA: African American
SPME: Solid phase microextraction
GC–MS: Gas chromatography-mass spectrometry
ROS: Reactive oxygen species
CYP450: Cytochrome P450
WHO: World health organization
CDC: Centers for disease control and prevention
