# Supplementary figures and images for: Urinary volatile organic compounds as potential non-invasive markers for childhood obesity
Source: Metabolomics. 2026 Jul 1;22(4):107. doi: 10.1007/s11306-026-02494-6 (PMC13323280; doi:10.1007/s11306-026-02494-6)

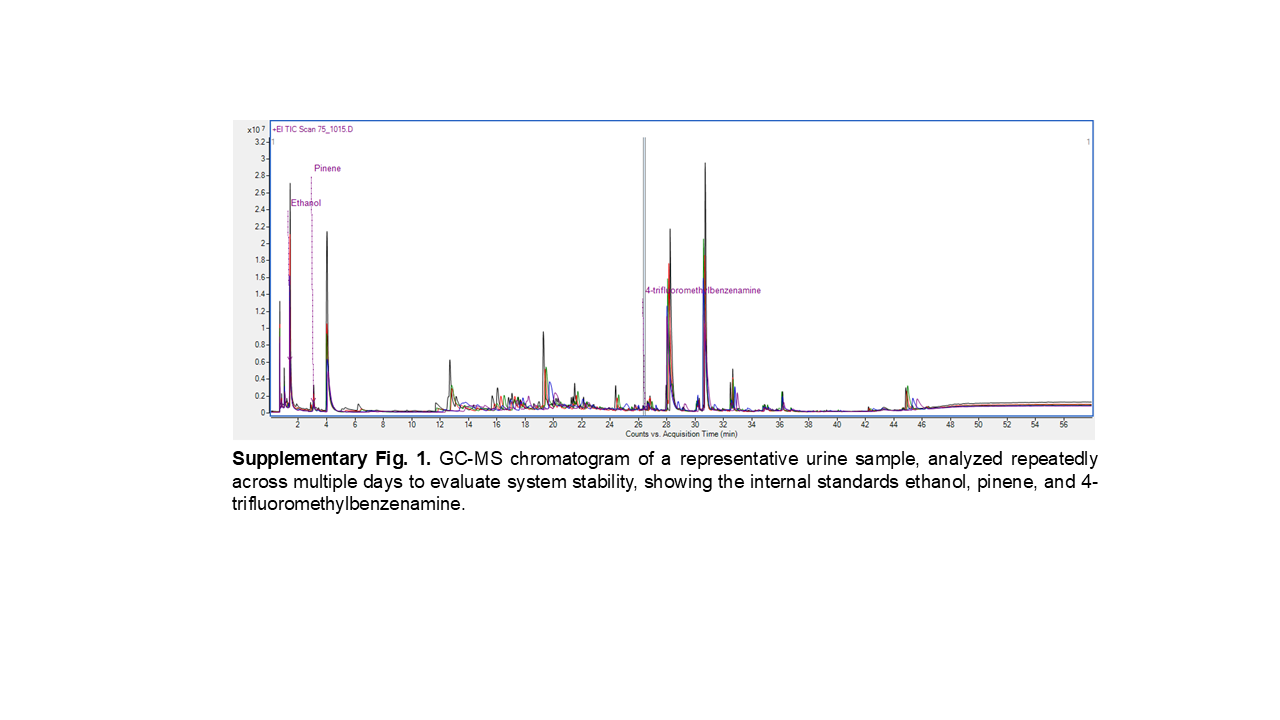

Supplement: Supplementary file 1 — Supplementary file1 (TIF 143 kb) [file 11306_2026_2494_MOESM1_ESM.tif]

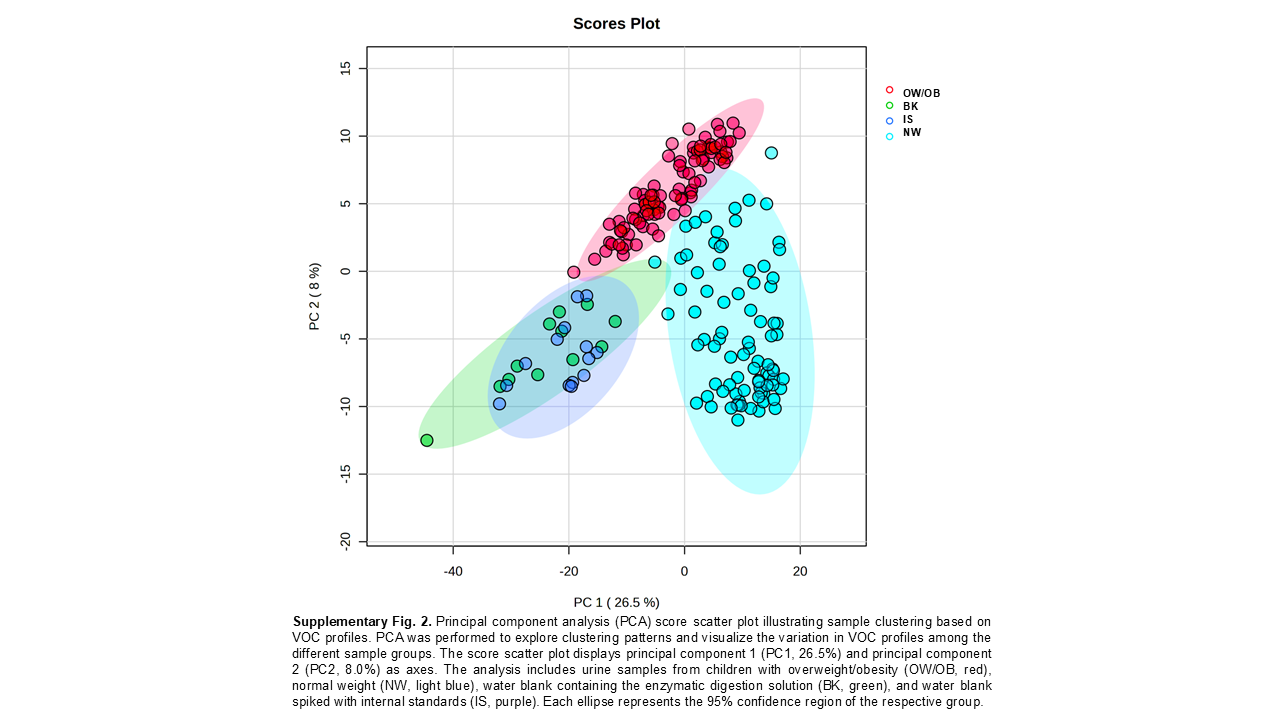

Supplement: Supplementary file 2 — Supplementary file2 (TIF 200 kb) [file 11306_2026_2494_MOESM2_ESM.tif]

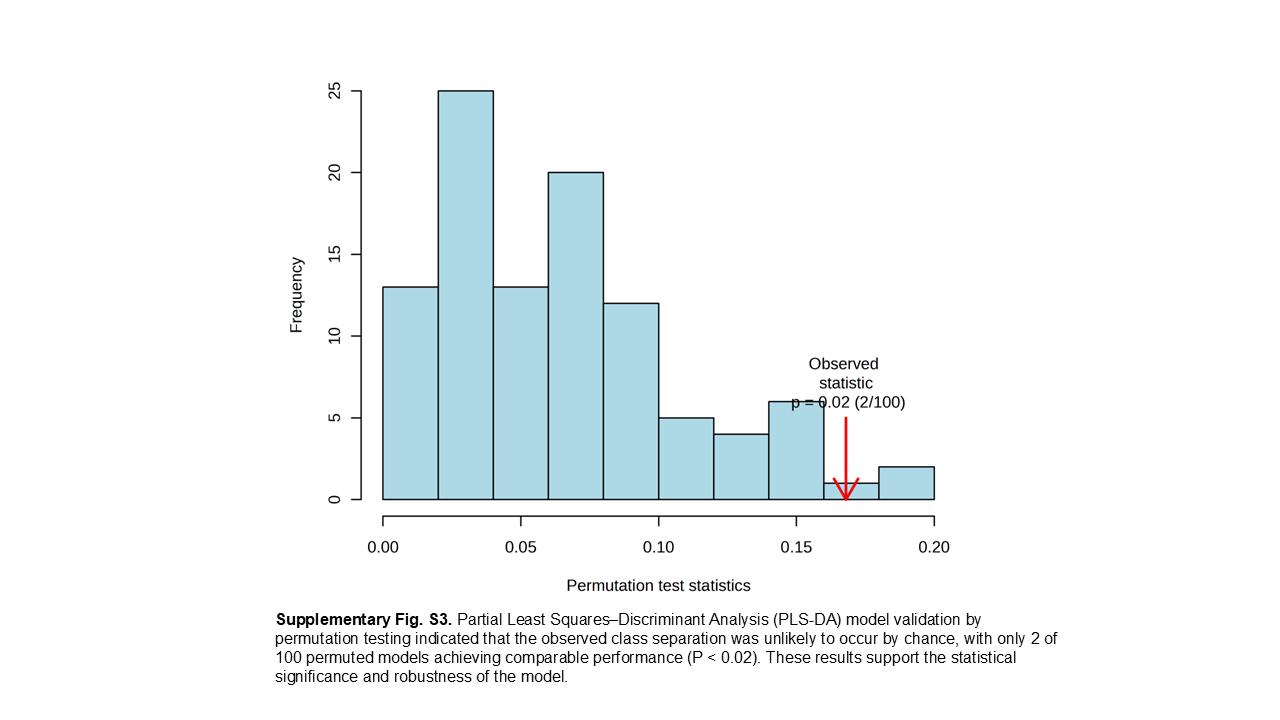

Supplement: Supplementary file 3 — Supplementary file3 (TIF 124 kb) [file 11306_2026_2494_MOESM3_ESM.tif]

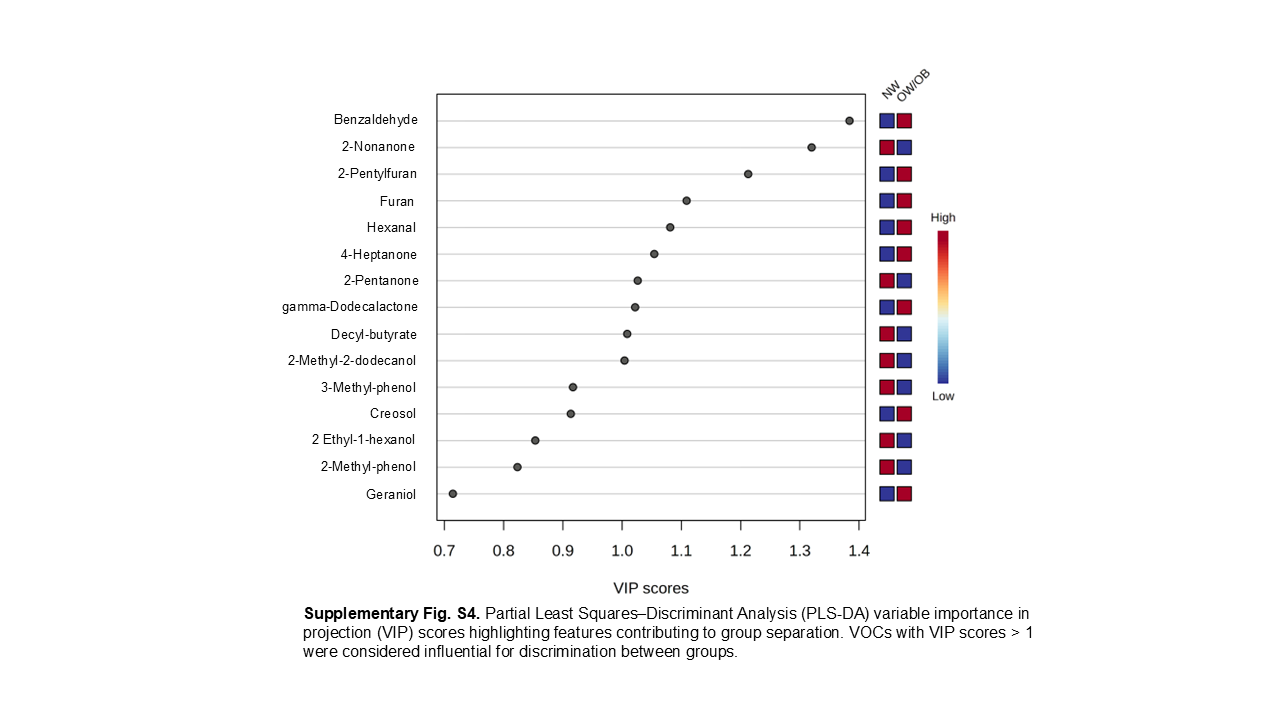

Supplement: Supplementary file 4 — Supplementary file4 (TIF 111 kb) [file 11306_2026_2494_MOESM4_ESM.tif]

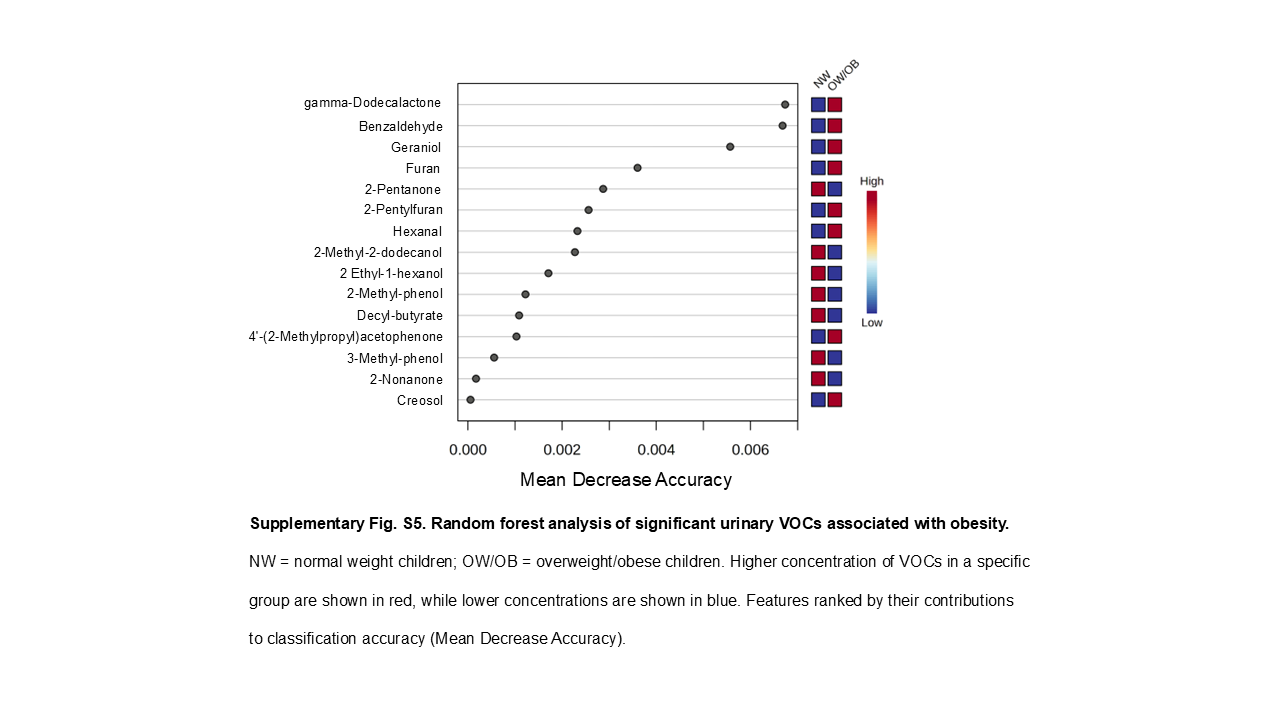

Supplement: Supplementary file 5 — Supplementary file5 (TIF 116 kb) [file 11306_2026_2494_MOESM5_ESM.tif]
