# Supplementary material for: Urinary volatile organic compounds as potential non-invasive markers for childhood obesity
Source: Metabolomics. 2026 Jul 1;22(4):107. doi: 10.1007/s11306-026-02494-6 (PMC13323280; doi:10.1007/s11306-026-02494-6)
Supplement: Supplementary file 6 — Supplementary file6 (DOCX 75 kb) [file 11306_2026_2494_MOESM6_ESM.docx]

**Supplement Table 1. Percentage of urinary VOCs occurrence in the normal weight and overweight/obese children**

| **Compounds** | **CAS** | **RT** | **Cal RI** | **Purity (%)** | **Ion (m/z)** | **Percentage of occurrence (%)** | |
| --- | --- | --- | --- | --- | --- | --- | --- |
|  |  |  |  |  |  | **NW** | **OW/OB** |
| **Ketones** |  |  |  |  |  |  |  |
| Acetone ^c^ | 67641 | 1.0353 |  | 92 | 43 | 100 | 100 |
| 2-Butanone | 78933 | 1.3356 |  | 23 | 43 | 78 | 64 |
| 2-Pentanone ^c^ | 107879 | 1.876 |  | 42 | 43 | 82 | 74 |
| 4-Heptanone ^c^ | 123193 | 4.0271 |  | 91 | 71 | 99 | 98 |
| 2-Methyl-3-hexanone | 7379126 | 4.1789 |  | 16 | 43 | 21 | 21 |
| 4'-(2-Methylpropyl) acetophenone | 38861788 | 26.6736 | 1719 | 89 | 116 | 4 | 5 |
| Oxybenzone | 131577 | 46.4488 |  | 12 | 227 | 97 | 89 |
| 2-Nonanone | 821556 | 14.3201 | 1231 | 48 | 132 | 83 | 90 |
| gamma-Dodecalactone ^c^ | 2305057 | 34.8029 | 1689 | 22 | 136 | 49 | 44 |
| 2-Heptanone ^c^ | 110430 | 6.2901 |  | 31 | 43 | 63 | 57 |
| 5-Tridecanone | 30692161 | 25.7725 | 1683 | 29 | 141 | 32 | 20 |
| 9-Heptadecanone | 540089 | 25.7725 | 1683 | 29 | 141 | 29 | 16 |
| (4-Chlorophenyl) phenyl-methanone | 134850 | 40.4123 |  | 35 | 216 | 6 | 16 |
| 3-Methyl-2-butanone | 563804 | 1.9007 |  | 17 | 109 | 4 | 10 |
| 2,4-Dimethyl-3-pentanone | 565800 | 2.0003 |  | 5.40 | 71 | 2 | 3 |
| 4-Hydroxy-2-methylacetophenone | 875592 | 31.9865 | 1966 | 21 | 107 | 12 | 11 |
| 1-(2,6,6-trimethyl-1,3-cyclohexadien-1-yl)-2-Buten-1-one | 14901076 | 18.6328 | 1396 | 9.70 | 177 | 22 | 25 |
| trans-3-Nonen-2-one | 18402830 | 17.0414 | 1333 | 44 | 55 | 13 | 10 |
| 3-Nonen-2-one | 14309570 | 17.0414 | 1333 | 44 | 55 | 15 | 11 |
| 4-Acetonylcycloheptanone | 86428606 | 19.421 | 1427 | 19 | 67 | 0 | 2 |
| **Hydrocarbon** |  |  |  |  |  |  |  |
| 2-Methyl-1-propene | 115117 | 0.7149 |  | 16 | 41 | 12 | 5 |
| 1-Butene | 106989 | 0.7141 |  | 11 | 41 | 23 | 23 |
| 2-Methyl-1-pentene | 763291 | 0.7749 |  | 13 | 56 | 38 | 43 |
| trans-1-Phenyl-1-pentene | 16002930 | 13.001 | 1154 | 40 | 131 | 8 | 13 |
| 1,3,5-Undecatriene | 19883295 | 14.5932 | 1241 | 32 | 91 | 32 | 43 |
| 1-(1,5-Dimethyl-4-hexenyl)-4-methylbenzene | 644304 | 22.9047 | 1560 | 42 | 119 | 23 | 15 |
| 2-Ethoxy-2-methylpropane | 637923 | 0.8134 |  | 59 | 59 | 92 | 93 |
| Methyl-cyclopentane | 96377 | 0.7863 |  | 3.70 | 56 | 5 | 7 |
| 1-Pentenyl-benzene | 826186 | 13.1598 | 1160 | 31 | 115 | 73 | 72 |
| 3-Cyclohexyl-1-phenyl-propane | 1.71E+08 | 36.4455 |  | 19 | 91 | 30 | 38 |
| Pentane | 109660 | 0.7191 |  | 9.90 | 43 | 23 | 30 |
| 2,4-Dimethyl-1-heptene | 19549872 | 1.2214 |  | 2.80 | 70 | 7 | 3 |
| 7-Ethyl-1,3,5-cycloheptatriene | 17634514 | 35.9302 |  | 12 | 91 | 6 | 11 |
| (2-Cyclopropylethenyl)-benzene | EPA-142016 | 17.0649 | 1334 | 50 | 91 | 33 | 21 |
| Cyclohexane | 110827 | 0.7749 |  | 13 | 56 | 26 | 16 |
| (4-Methyl-1-methylenepent-4-enyl) benzene | 63942881 | 31.3584 | 1935 | 32 | 129 | 6 | 3 |
| 1-Nitro-heptane | 693390 | 18.808 | 1403 | 12 | 55 | 10 | 7 |
| 2-Butene | 107017 | 0.7149 |  | 16 | 41 | 10 | 5 |
| **Aldehyde** |  |  |  |  |  |  |  |
| Benzaldehyde ^c^ | 100527 | 16.7832 | 1322 | 83 | 78 | 62 | 61 |
| 3,5-Di-tert-butyl-4-hydroxybenzaldehyde | 1620980 | 36.0827 |  | 13 | 219 | 26 | 23 |
| 3-Methyl-benzaldehyde | 620235 | 20.5399 | 1463 | 33 | 120 | 11 | 10 |
| 2-Methyl-benzaldehyde | 529204 | 20.5399 | 1463 | 33 | 120 | 33 | 25 |
| 4-Methyl-benzaldehyde | 104870 | 20.5399 | 1463 | 33 | 120 | 9 | 10 |
| Acetaldehyde ^c^ | 75070 | 0.8376 |  | 51 | 43 | 98 | 100 |
| 2-Ethyl-hexanal | 123057 | 12.0429 | 1100 | 11 | 43 | 5 | 11 |
| Hexanal ^c^ | 66251 | 3.3078 |  | 30 | 72 | 47 | 56 |
| Pentanal | 110623 | 1.3772 |  | 11 | 39 | 4 | 7 |
| Pentadecanal | 2765119 | 30.479 | 1888 | 18 | 57 | 43 | 49 |
| 2-Methyl-butanal | 96173 | 1.3734 |  | 11 | 39 | 4 | 3 |
| 2-Methyl-3-phenyl-2-propenal | 101393 | 21.9877 | 1516 | 14 | 145 | 1 | 7 |
| 2-Methyl-propanal | 75650 | 1.2539 |  | 12 | 59 | 8 | 7 |
| **Nitrogen, Sulphur, Ether containing compound** |  |  |  |  |  |  |  |
| 3-Amino-5-t-butylisoxazole ^c^ | 55809364 | 3.3953 |  | 6.40 | 115 | 3 | 2 |
| Methanethiol | 74931 | 0.8064 |  | 16 | 47 | 31 | 43 |
| Dimethyl disulfide | 624920 | 2.917 |  | 16 | 46 | 16 | 13 |
| Salicylaldoxime | 94-67-7 | 23.2929 | 1583 | 38 | 69 | 37 | 36 |
| 2,2,4-Trimethyl-1,3-pentanediol diisobutyrate | 6846500 | 25.2144 | 1660 | 59 | 71 | 91 | 89 |
| N-(2-Hydroxyethyl)-N-methyl-perfluorobutane-1-sulfonamide | EPA-510211 | 26.7185 | 1721 | 26 | 326 | 83 | 85 |
| 8-Propoxy-cedrane | 19870758 | 29.3532 | 1856 | 21 | 150 | 3 | 3 |
| Homosalate | 118569 | 35.7482 |  | 8.30 | 190 | 80 | 69 |
| 3-Tert-butyl-4-hydroxyanisole | 121006 | 36.1455 |  | 31 | 165 | 43 | 54 |
| 7,7-Dimethyl-5,6,7,8-tetrahydro-thiazolo[5,4-c] azepine-4-thione | EPA-318052 | 43.0825 |  | 21 | 197 | 17 | 18 |
| Ethyl-pyrazine | 13925003 | 27.9055 | 1350 | 5.00 | 107 | 5 | 8 |
| 5H-1-Pyrindine | 270917 | 34.6364 |  | 74 | 117 | 15 | 15 |
| 1,6-Dimethyl-4-(1-methylethyl)-naphthalene | 483783 | 31.6074 | 1962 | 36 | 183 | 26 | 18 |
| 1,4-Dimethyl-7-(1-methylethyl)-azulene | 489849 | 31.6074 | 1962 | 36 | 183 | 27 | 20 |
| Cyclohexyl salicylate | EPA-431586 | 33.6578 |  | 59 | 120 | 7 | 18 |
| 2,5-Dimethylhexane-2,5-dihydroperoxide | 3025885 | 5.2804 |  | 10 | 43 | 6 | 5 |
| Alanine | 56417 | 2.6911 |  | 16 | 44 | 20 | 11 |
| Methyl anthranilate | 134203 | 31.7177 | 1952 | 62 | 119 | 10 | 13 |
| Alpha, alpha, 4-trimethyl-, (R)- 3-cyclohexene-1-methanol | 7785537 | 21.1737 | 1487 | 21 | 59 | 8 | 5 |
| **Alcohols** |  |  |  |  |  |  |  |
| Alpha-terpineol | 10482561 | 21.0459 | 1480 | 38 | 81 | 31 | 25 |
| p-Mentha-1,5-dien-8-ol | 1686200 | 21.5999 | 1499 | 18 | 94 | 40 | 30 |
| Mequinol | 150765 | 24.3776 | 1628 | 3.40 | 124 | 50 | 56 |
| 2-Methoxy-phenol | 90051 | 24.3776 | 1632 | 3.40 | 124 | 86 | 85 |
| Creosol ^c^ | 93516 | 26.0862 | 1696 | 67 | 123 | 80 | 69 |
| 2-Methoxy-5-methylphenol | 1195091 | 26.0862 |  | 67 | 123 | 79 | 70 |
| Phenol | 108952 | 26.5306 | 1714 | 87 | 94 | 98 | 97 |
| p-Cresol ^c^ | 106445 | 27.7883 | 1778 | 97 | 107 | 98 | 100 |
| 3-Methyl-phenol ^c^ | 108394 | 27.7883 | 1778 | 97 | 107 | 100 | 98 |
| 2-Methyl-phenol ^c^ | 95487 | 28.0248 | 1789 | 2.90 | 107 | 39 | 41 |
| 3,7,11-Trimethyl-2,6,10-dodecatrien-1-ol | 3790714 | 33.1377 |  | 30 | 93 | 39 | 54 |
| 4-Ethyl-phenol | 123079 | 30.0338 | 1888 | 85 | 107 | 52 | 52 |
| Eugenol | 97530 | 30.1284 | 1871 | 70 | 164 | 64 | 77 |
| 3-Ethyl-phenol | 620177 | 30.1661 | 1878 | 11 | 107 | 14 | 25 |
| Thymol | 89838 | 30.3839 | 1885 | 2.10 | 135 | 55 | 56 |
| 2-Methyl-5-(1-methylethyl)-phenol | 499752 | 30.8466 | 1926 | 70 | 135 | 77 | 77 |
| 2,5-Dichloro-phenol | 583788 | 31.2508 | 1930 | 9.70 | 162 | 31 | 13 |
| 2-Methoxy-4-vinylphenol | 7786610 | 31.3204 | 1948 | 21 | 151 | 70 | 61 |
| 5-Ethenyl-2-methoxy-phenol | 621589 | 31.3204 |  | 21 | 151 | 26 | 31 |
| 4-Vinylphenol | 2628173 | 34.387 |  | 40 | 119 | 67 | 64 |
| Chloroxylenol | 88040 | 37.4807 |  | 28 | 156 | 13 | 10 |
| 2-Chloro-4-methyl-phenol | 6640273 | 25.8745 | 1715 | 33 | 77 | 38 | 38 |
| 3-Chloro-4-methyl-phenol | 615623 | 25.8745 | 1715 | 33 | 77 | 22 | 23 |
| 2-Methyl-2-propanol | 75650 | 1.2521 |  | 28 | 59 | 62 | 49 |
| 2,6-Dimethyl-2-octanol | 18479588 | 16.0007 | 1292 | 44 | 59 | 30 | 31 |
| 3-Methyl-1-dodecyn-3-ol | 24424780 | 26.8739 | 1733 | 40 | 84 | 32 | 34 |
| trans-Farnesol ^c^ | 106285 | 33.8532 |  | 31 | 69 | 17 | 15 |
| 1-Octen-3-ol ^c^ | 3391864 | 14.7073 | 1245 | 29 | 57 | 38 | 21 |
| 2-Ethyl-1-hexanol ^c^ | 104767 | 16.0611 | 1294 | 34 | 41 | 83 | 79 |
| 3,5,5-Trimethyl-1-hexanol | 3452979 | 16.2832 | 1302 | 4.80 | 56 | 9 | 8 |
| 3,3,5-Trimethyl-, cis-cyclohexanol | 933482 | 17.3375 | 1345 | 15 | 109 | 17 | 21 |
| 2-(1,1-Dimethylethyl)-cyclohexanol | 13491797 | 19.0628 | 1412 | 12 | 57 | 15 | 31 |
| 2-Undecanol | 1653301 | 21.7234 | 1504 | 37 | 45 | 10 | 8 |
| 1-Decanol | 112301 | 22.347 | 1534 | 10 | 56 | 33 | 25 |
| 2,6-Bis(1,1-dimethylethyl)-phenol | 128392 | 23.9054 | 1605 | 19 | 191 | 37 | 21 |
| Geraniol ^c^ | 106241 | 24.1144 | 1613 | 25 | 69 | 20 | 23 |
| 4,6-Di(1,1-dimethylethyl)-2-methyl-phenol | 616557 | 25.8968 | 1716 | 3.30 | 205 | 37 | 36 |
| 6,10-Dimethyl-5,9-undecadien-2-ol | 53837346 | 26.7925 | 1730 | 54 | 109 | 39 | 33 |
| 4-Ethyl-2-methoxy-phenol | 2785899 | 27.2903 | 1754 | 10 | 152 | 56 | 46 |
| 3,7,11-Trimethyl-1,6,10-dodecatrien-3-ol | 40716663 | 28.5954 | 1817 | 27 | 81 | 72 | 57 |
| 4-(2-Propenyl)-phenol | 501928 | 36.8823 |  | 9.50 | 134 | 28 | 25 |
| 4,6-Di-tert-butyl-m-cresol | 497392 | 38.1225 |  | 31 | 205 | 14 | 11 |
| 2-Benzylidene-1-heptanol | 101859 | 38.3761 |  | 46 | 133 | 63 | 59 |
| 4-(7-Methyloctyl) phenol | 24518487 | 38.8578 |  | 12 | 135 | 37 | 30 |
| Alpha-phenyl-benzenemethanol | 91010 | 39.7817 |  | 9.40 | 105 | 15 | 13 |
| 1-Nonanol ^c^ | 143088 | 20.0073 | 1445 | 41 | 70 | 48 | 48 |
| 2-Propyl-1-heptanol | 10042598 | 20.1867 | 1453 | 58 | 98 | 22 | 20 |
| 3-Nonen-1-ol | 10340235 | 20.3327 | 1456 | 25 | 68 | 29 | 21 |
| 11,11-Dimethyl-4,8-dimethylenebicyclo [7.2.0] undecan-3-ol | 79580011 | 32.9636 |  | 10 | 136 | 7 | 3 |
| 9-Phenyl-n-nonanol | EPA-342461 | 36.4455 |  | 19 | 91 | 18 | 10 |
| 2-Propyl-1-pentanol | 58175578 | 16.0611 | 1294 | 34 | 41 | 35 | 28 |
| 1,7,7-Trimethylbicyclo [2.2.1] heptan-2-ol | 10385781 | 20.2322 | 1453 | 40 | 95 | 6 | 2 |
| 2-Allyl-4-methylphenol | 6628064 | 34.7977 |  | 30 | 148 | 7 | 3 |
| 2,6-Dimethyl-7-octen-2-ol | 18479588 | 15.6551 | 1278 | 21 | 59 | 32 | 31 |
| 2,5-Dimethyl-2,5-hexanediol | 110032 | 1.135 |  | 5.10 | 113 | 13 | 16 |
| 3,7-Dimethyl-2,6-octadien-1-ol | 624157 | 24.1144 | 1613 | 25 | 69 | 21 | 13 |
| 3-Methyl-4-(methylthio)-phenol | 3120749 | 27.4283 | 1760 | 12 | 154 | 12 | 2 |
| p-Tert-butyl-phenol | 98544 | 32.1704 | 1976 | 36 | 135 | 7 | 3 |
| m-Tert-butyl-phenol | 585342 | 32.091 | 1973 | 5.60 | 135 | 4 | 7 |
| 4-Pentyl-phenol | 14938353 | 16.4261 | 1308 | 15 | 107 | 30 | 43 |
| 2-Methoxy-4-propyl-phenol ^c^ | 2785877 | 29.0518 | 1839 | 18 | 137 | 7 | 13 |
| 2-Methoxy-4-(1-propenyl)-phenol | 5912867 | 34.0088 |  | 52 | 164 | 12 | 10 |
| 2-Methyl-6-(p-tolyl) hept-2-en-4-ol | 38142573 | 25.4231 | 1691 | 37 | 119 | 10 | 7 |
| 5-Methyl-2-(6-methylhept-5-en-2-yl) phenol | 69301275 | 38.2403 |  | 39 | 135 | 11 | 8 |
| 5-(1,5-Dimethyl-4-hexenyl)-2-methylphenol | 30199269 | 39.4186 |  | 17 | 136 | 10 | 5 |
| p-Mentha-1,8-dien-7-ol | 536594 | 27.1526 | 1747 | 17 | 79 | 7 | 15 |
| 4-(1,1-dimethylpropyl)-phenol | 80466 | 34.1631 |  | 18 | 135 | 14 | 23 |
| (-)-Trans-Isopiperitenol | 74410007 | 22.0538 | 1518 | 9.30 | 84 | 5 | 7 |
| 2-Methyl-2-decanol | 3396029 | 15.044 | 1256 | 14 | 59 | 10 | 11 |
| 3-Tridecanol | 10289686 | 16.6382 | 1316 | 28 | 59 | 6 | 15 |
| 3,7-Dimethyl-3-octanol | 78693 | 15.3388 | 1266 | 19 | 73 | 34 | 30 |
| 3-Tetradecanol | 1653323 | 19.0636 | 1412 | 16 | 59 | 3 | 3 |
| 2-Methyl-2-dodecanol | 1653378 | 19.0636 | 1412 | 16 | 59 | 4 | 2 |
| **Acids and Esters** |  |  |  |  |  |  |  |
| Benzoic acid, 2-hydroxy-, ethyl ester | 2050080 | 30.0063 | 1887 | 5.90 | 120 | 46 | 54 |
| Propanoic acid, 2-methyl-, 3-hydroxy-2,2,4-trimethylpentyl ester | 77689 | 24.5422 | 1633 | 28 | 145 | 19 | 33 |
| 2-Ethyl-hexanoic acid | 149575 | 25.5829 | 1703 | 12 | 88 | 22 | 18 |
| Decyl-butyrate | 108-32-7 | 25.6789 | 1707 | 43 | 71 | 13 | 20 |
| (p-Hydroxyphenyl)- phosphonic acid | 33795185 | 26.6064 | 1724 | 26 | 94 | 88 | 85 |
| 2-Decenoic acid | 3913857 | 32.4431 | 1989 | 84 | 55 | 19 | 34 |
| Trans-2-decenoic acid | 334496 | 32.4431 | 1989 | 84 | 55 | 33 | 38 |
| 9-Octadecenoic acid, ethyl ester | 111615 | 36.2424 |  | 13 | 88 | 53 | 56 |
| 2-Nonenoic acid | 3760110 | 30.5288 | 1913 | 31 | 73 | 5 | 13 |
| Octadecanoic acid, ethyl ester | 111615 | 36.3242 | 1700 | 13 | 88 | 63 | 59 |
| Carbamic acid, methyl-, 3-methylphenyl ester | 1129415 | 28.5372 | 1814 | 2.10 | 216 | 4 | 3 |
| Hexadecanoic acid, ethyl ester | 628977 | 33.7335 | 1600 | 19 | 88 | 95 | 92 |
| Orthoformic acid, triisobutyl ester | 16754497 | 4.6982 |  | 18 | 57 | 72 | 66 |
| 2-Thiophenecarboxylic acid, 4-nitrophenyl ester | EPA-308067 | 27.0964 | 1744 | 2.80 | 111 | 36 | 25 |
| 2-Octylcyclopropane-1-carboxylic acid | EPA-486867 | 30.5676 | 1915 | 17 | 41 | 10 | 13 |
| 3-Octenoic acid | 1577191 | 29.0753 | 1840 | 42 | 41 | 14 | 21 |
| n-Decanoic acid | 334485 | 30.5496 | 1914 | 19 | 73 | 32 | 34 |
| Geranic acid | 459803 | 33.1737 |  | 31 | 69 | 6 | 10 |
| Neric acid | 4613381 | 32.2567 | 1980 | 4.40 | 100 | 3 | 3 |
| 2-Methyl-octanoic acid | 3004931 | 27.2176 | 1750 | 49 | 74 | 5 | 7 |
| cis-2-Octenoic acid | 1577964 | 29.0753 |  | 22 | 41 | 7 | 11 |
| o-Aminobenzohydroxamic acid | 5623041 | 23.4906 | 1587 | 34 | 120 | 3 | 3 |
| 3,7-Dimethyl-2,6-octadienoic acid | 4698082 | 33.1737 |  | 31 | 69 | 10 | 8 |
| **Terpenes** |  |  |  |  |  |  |  |
| alpha-Calacorene | 21391991 | 25.2482 | 1684 | 52 | 157 | 39 | 25 |
| alpha-Corocalene | 20129399 | 28.4236 | 1808 | 2.30 | 185 | 10 | 15 |
| Indole ^c^ | 120729 | 34.741 |  | 65 | 90 | 87 | 85 |
| t-Butylhydroquinone | 1948330 | 45.1968 |  | 4.90 | 123 | 58 | 54 |
| dl-Menthol | 89781 | 19.2422 | 1419 | 60 | 95 | 72 | 74 |
| Linalool | 78706 | 17.4841 | 1350 | 17 | 93 | 65 | 46 |
| Terpinen-4-ol | 562743 | 18.6224 | 1396 | 13 | 71 | 24 | 18 |
| (-)-Carvone | 6485401 | 21.9944 | 1516 | 87 | 82 | 6 | 10 |
| D-Carvone | 2244168 | 22.0317 | 1517 | 12 | 82 | 11 | 5 |
| Indolizine | 274408 | 34.792 |  | 46 | 90 | 22 | 18 |
| 10,11-Epoxycalamenene | 1.44E+08 | 24.7719 | 1655 | 6.80 | 173 | 19 | 11 |
| Butylated hydroxytoluene | 128370 | 25.0186 | 1672 | 33 | 205 | 69 | 62 |
| Erucin | 4430368 | 29.8713 | 1881 | 10 | 115 | 4 | 5 |
| 5-Acetyl-4-amino-2-methylthio-thiophene-3-carbonitrile | 1.16E+08 | 46.3576 |  | 10 | 212 | 19 | 23 |
| endo-Borneol | 507700 | 20.2079 | 1452 | 40 | 95 | 27 | 16 |
| Heptaethylene glycol | 5617323 | 54.507 |  | 1.90 | 110 | 30 | 23 |
| trans-Verbenol | 1820093 | 20.0985 | 1448 | 22 | 109 | 5 | 7 |
| (-)-Myrtenol | 19894974 | 22.5279 | 1539 | 6.00 | 128 | 9 | 3 |
| trans-Calamenene | 73209424 | 23.8172 | 1600 | 2.40 | 159 | 23 | 16 |
| Nerolidol | 142507 | 28.2681 | 1800 | 21 | 67 | 14 | 7 |
| 2-(sec-Butyl amino)-benzoxazole | 28291829 | 23.1986 | 1574 | 19 | 219 | 5 | 5 |
| Carveol | 99489 | 24.3053 | 1627 | 77 | 84 | 2 | 7 |
| Trans-carveol | 1197075 | 23.7833 | 1599 | 52 | 109 | 11 | 10 |
| Alpha-dehydro-ar-himachalene | 78204623 | 25.0988 | 1677 | 26 | 157 | 5 | 3 |
| 4,5,9,10-Dehydro-isolongifolene | 1.57E+08 | 25.0988 | 1677 | 26 | 157 | 4 | 5 |
| (Trimethylsilyl)-ferrocene | 12215688 | 40.2759 |  | 13 | 243 | 5 | 8 |
| cis-Calamenene | 72937554 | 27.0432 | 1741 | 27 | 202 | 4 | 10 |
| alpha-Cadinol | 481345 | 30.714 | 1922 | 18 | 95 | 6 | 5 |
| Ylangenol | 41610699 | 32.7206 |  | 24 | 177 | 7 | 3 |
| tau-Muurolol | 19912620 | 30.714 | 1922 | 18 | 95 | 4 | 3 |
| beta-Farnesene | 18794848 | 21.1398 | 1486 | 34 | 69 | 8 | 5 |
| **Furans** |  |  |  |  |  |  |  |
| 2,3-Dihydro-benzofuran | 496162 | 34.5147 |  | 23 | 120 | 15 | 26 |
| Furan ^c^ | 110009 | 0.9562 |  | 18 | 68 | 65 | 69 |
| 2-Methyl-furan | 534225 | 1.1802 |  | 17 | 82 | 15 | 18 |
| 3-Methyl-furan | 930278 | 1.1802 |  | 17 | 82 | 76 | 79 |
| 5-Dodecyldihydro-2(3H)-furanone | 730461 | 41.6698 |  | 8.10 | 85 | 4 | 10 |
| 5-Heptyldihydro-2(3H)-furanone | 104676 | 30.2677 | 1900 | 2.90 | 85 | 11 | 13 |
| Dihydro-5-pentyl-2(3H)-furanone | 104610 | 30.2677 | 1900 | 2.90 | 85 | 6 | 5 |
| 2-Pentyl-furan | 3777693 | 7.5337 |  | 11 | 138 | 31 | 33 |
| 2-Ethyl-5-methyl-furan | 1703522 | 2.3779 |  | 12 | 95 | 57 | 75 |
| 2,5-Dimethyl-furan | 625865 | 1.6737 |  | 10 | 95 | 42 | 36 |
| 4,7-Dimethyl-benzofuran | 28715266 | 22.0305 | 1517 | 15 | 115 | 18 | 18 |

m/z: mass-to-charge ratio; RT: retention time, RI: retention index, Purity: NIST library spectra match, ^c^: confirmed by reference standard; CAS: chemical abstracts service, NW: normal weight children; OW/OB: overweight/obese children.
