# Supplementary material for: Urinary volatile organic compounds as potential non-invasive markers for childhood obesity
Source: Metabolomics. 2026 Jul 1;22(4):107. doi: 10.1007/s11306-026-02494-6 (PMC13323280; doi:10.1007/s11306-026-02494-6)
Supplement: Supplementary file 7 — Supplementary file7 (DOCX 22 kb) [file 11306_2026_2494_MOESM7_ESM.docx]

**Supplement Table 2. The association between urinary VOCs and BMI z-score using logistic regression**

| **VOCs** | **Unadjusted** | | | **Model 1** | | | **Model 2** | | |
| --- | --- | --- | --- | --- | --- | --- | --- | --- | --- |
|  | **B** | **OR (95%CI)** | ***P***  **value** | **B** | **OR (95%CI)** | ***P***  **value** | **B** | **OR (95%CI)** | ***P***  **value** |
| Furan | 25.15 | 8.39E+10  (189.97 – 3.70E+19) | 0.013 | 25.24 | 9.16E+10  (200.92 – 4.18E+19) | 0.013 | 24.81 | 5.96E10  (96.79 – 3.66E+19) | 0.016 |
| Hexanal | 65.05 | 1.78E+28  (47944.19 – 6.69E+51) | 0.019 | 64.97 | 1.64E+28  (42379.49 – 6.38E+51) | 0.019 | 68.70 | 6.85E+29  (267084.03 – 1.76E+54) | 0.017 |
| 4-Heptanone | 0.37 | 1.44 (1.056 – 1.975) | 0.022 | 0.38 | 1.46 (1.058 – 2.0) | 0.021 | 0.33 | 1.394 (1.002 – 1.939) | 0.049 |
| 2-Pentylfuran | 34.27 | 7.66E+14  (8605.57 – 6.81E+25) | 0.008 | 34.28 | 7.70E+14  (8430.92 – 7.04E+25) | 0.008 | 33.75 | 4.56E+14  (4129.43 – 5.03E+25) | 0.009 |
| Benzaldehyde | 21.72 | 2.70E+9  (182.93 – 3.98E+16) | 0.010 | 21.78 | 2.88E+9  (185.32 – 4.46E+16) | 0.010 | 21.09 | 1.45E+9  (68.50 – 3.05E+16) | 0.014 |
| 4'-(2-Methylpropyl) acetophenone | 320.92 | 2.36E+139  (3.04E+28 – 1.83E+250) | 0.014 | 326.09 | 4.18E+141  (1.67E+29 – 1.05E+254) | 0.014 | 307.20 | 2.59E+133  (4.0E+19 – 1.68E+247) | 0.022 |
| Creosol | 16.10 | 9.83E+6  (0.574 – 1.68E+14) | 0.058 | 16.65 | 1.71E+7  (0.759 – 3.84E+14) | 0.054 | 17.38 | 3.54E+7  (1.56 – 8.03E+14) | 0.044 |
| gamma-Dodecalactone | 51.09 | 1.53E+22  (278.41 – 8.46E+41) | 0.028 | 51.03 | 1.45E+22  (235.16 – 8.90E+41) | 0.028 | 60.18 | 1.36E+26  (3.46E+5 – 5.37E+46) | 0.013 |
| 2-Nonanone | -15.92 | 0.00  (0.00 – 0.216) | 0.030 | -16.12 | 0.00  (0.00 – 0.194) | 0.029 | -15.28 | 0.00  (0.00 – 0.220) | 0.030 |
| 2 Ethyl-1-hexanol | -3.28 | 0.038  (0.001 – 1.142) | 0.060 | -3.35 | 0.035  0.001 – 1.074) | 0.055 | -3.26 | 0.039  (0.001 – 1.278) | 0.068 |
| 2-Methyl-2-dodecanol | -6.98 | 0.001  (0.00 – 0.492) | 0.029 | -6.98 | 0.001  (0.00 – 0.495) | 0.029 | -7.16 | 0.001  (0.00 – 0.489) | 0.029 |
| Decyl-butyrate | -0.76 | 0.467  (0.236 – 0.921) | 0.028 | -0.76 | 0.466  (0.237 – 0.919) | 0.028 | -0.79 | 0.452  (0.222 – 0.919) | 0.028 |
| 3-Methyl-phenol | -0.04 | 0.962  (0.927 – 0.998) | 0.037 | -0.039 | 0.962  (0.927 – 0.998 | 0.037 | -0.04 | 0.960  (0.925 – 0.997) | 0.032 |
| 2-Methyl-phenol | -3.14 | 0.043  (0.002 – 1.164) | 0.062 | -3.193 | 0.041  (0.002 – 1.122) | 0.058 | -3.08 | 0.046  (0.002 – 1.339) | 0.073 |

Unadjusted: model without any covariates; model 1: model adjusted by race; model 2: model adjusted by race, family income and maternal education. OR: Odds ratio; CI: confidence interval; B: model coefficients.
